# Phakellistatins: An Underwater Unsolved Puzzle

**DOI:** 10.3390/md15030078

**Published:** 2017-03-18

**Authors:** Alessandra Meli, Consiglia Tedesco, Giorgio Della Sala, Rosaria Schettini, Fernando Albericio, Francesco De Riccardis, Irene Izzo

**Affiliations:** 1 Department of Chemistry and Biology “A. Zambelli”, University of Salerno, Via Giovanni Paolo II, 132-84081 Fisciano (SA), Italy; ctedesco@unisa.it (C.T.); gdsala@unisa.it (G.D.S.); rschettini@unisa.it (R.S.); dericca@unisa.it (F.D.R.); 2 Department of Organic Chemistry and CIBER-BBN, Networking Centre on Bioengineering, Biomaterials and Nanomedicine, Barcelona Science Park, University of Barcelona, Barcelona 08028, Spain; albericio@ub.edu; 3 School of Chemistry and Physics, University of KwaZulu-Natal, Durban 4001, South Africa

**Keywords:** marine sponge, proline-rich cyclopeptide, peptide synthesis, pharmacological activity

## Abstract

A critical summary on the discovery of the nineteen members of the phakellistatin family (phakellistatin **1**–**19**), cytotoxic proline-rich cyclopeptides of marine origin, is reported. Isolation, structural elucidation, and biological properties of the various-sized natural macrocycles are described, along with the total syntheses and the enigmatic issues of the cytotoxic activity reproducibility.

## 1. Introduction

Marine organisms are an impetuous reservoir of intriguing natural products [1]. Over billions of years of evolution, the bioactivities of these secondary metabolites have been finely tuned and still, today, they represent an unrivaled source of inspiration for the discovery of new drugs: from antiviral, to antibiotic, to cytostatic, to antitumor qualities [2,3].

Among the many marine species, *Porifera*, commonly known as sponges, account for the less-evolved pluricellular phylum. Living in a highly-competitive environment, exposed to predators, they developed a sophisticated chemical defense system and remain the dominant reservoir of bioactive metabolites [1]. Although, more and more, the origin of these compounds appears to be endosymbiont-derived (microalgae, bacteria, archaea, fungi, cyanobacteria, producing nutrients which the sponge is not able to obtain through its own filter-feeding ability), the supply problem is still challenging and no sustainable production processes are available to date [4,5,6,7,8]. Peptides represent a wide subclass of natural bioactive compounds that, for diversity and simplicity of the structural units, encompasses all of the others. They are classified according to their biosynthetic pathways in ribosomal and non-ribosomal peptides (the last produced by large multienzyme complexes, such as non-ribosomal peptide synthetases (NRPS)) [9]. Cyclopeptides and cyclodepsipeptides, replacing at least one amide bond with an ester linkage, are the more promising lead structures [9,10,11]. The absence of the free polar C- and *N*-termini, drastically improves cellular uptake and resistance to enzymatic degradation [12]. Moreover, the reduced flexibility due to macrocyclization, heterocyclization, or cross-linking generally enhances active three-dimensional structures favoring specific binding with proper biological targets [13,14]. Another intriguing feature of cyclic peptides/depsipeptides is represented by the presence of unusual amino acids, such as β-hydroxylated, *N*-alkylated, d-amino acids, or heterocyclic moieties (oxazolines, oxazoles, thiazolines, and thiazoles, which come from amino acids, such as Ser/Thr and Cys), alternated within standard amino acid sequences. Such structural modifications contribute to modulate the biological functions and define specific pharmacological properties [15,16]. Since the first isolation of the anti-inflammatory cyclotetradecapeptide discodermin A from the sponge *Discodermia kiiensis*, in the 1980s by Fusetani and co-workers [17], the demand for sufficient amounts of such interesting compounds for further studies has become even more pressing. Unfortunately, because of the ambiguities about the actual biological source and their poor natural availability, the evaluation of the biological potential of these promising bioactive peptides frequently has to await the accomplishment of a suitable synthetic route. For these natural compounds the total synthesis still plays a central role in developing their therapeutic potential, also confirming the correct structural assignment of the isolated active compound [18]. Moreover, the development of an efficient total synthesis can allow the introduction of artificial modifications yielding more active analogues.

## 2. Phakellistatins: Isolation, Structure, and Properties

Proline-rich cyclopeptides belong to a captivating class of natural compounds with a wide range of biological functions. Some of them act as specific modulators of pharmacological targets and are used in the clinic (e.g., tyrocidine, gramicidin S) and clinical trials (e.g., aplidine). The Pro residue plays an important structural role in the macrocycle for its innate ability to reduce the conformational flexibility and induce specific conformations, which is often translated in an improved bioactivity [19].

**Figure 1:**
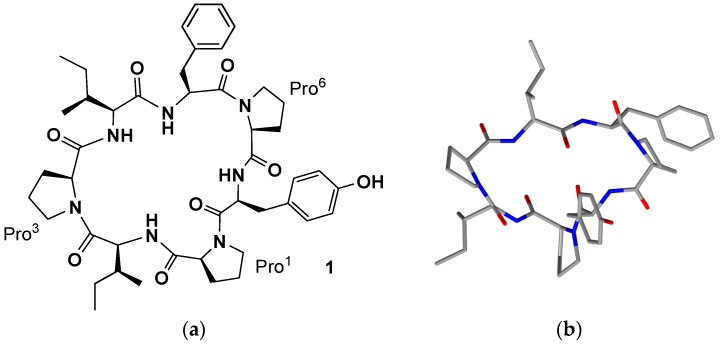
(**a**) Phakellistatin **1**; and (**b**) X-ray crystal structure of phakellistatin **1**.

Pettit et al. were, for a long time, especially interested into the sponges of the *Demospongia* class (including more than 10,000 species) [20]. Their studies led to the isolation of new cell growth inhibitory and antineoplastic substances from *Axinella* sp. (class *Demospongiae*, order *Axinellida*), *Stylotella aurantium* (class *Demospongiae*, order *Halichondrida*) and from *Phakellia* sp. (class *Demospongiae*, order *Axinellida*). In 1985, upon a return expedition to Palau in the Western Caroline Islands, they collected specimens of the sponge *S. aurantium*. As part of a 1987 exploratory survey of the Truk (or Chuuk) Archipelago (Federated States of Micronesia) they found a *Phakellia* species, *Phakellia costata* that, similarly to the *S. aurantium* afforded aqueous iPrOH extracts that significantly inhibited growth of the murine PS leukemia in vivo. Both *Phakellia* (family *Axinellidae*) and *Stylotella* (family *Hymeniacidonidae*) species, considered distantly related, were found to contain the same new PS leukemia cell line-growth-inhibitory cycloheptapeptide, named phakellistatin 1 (**1**, Table 1, Figure 1), whose presence suggested their probable monophyletic origin or the presence of a common symbiotic microorganism. The structure was determined using high-field NMR (500 MHz), amino acid analyses and mass spectral techniques (Fast Atom Bombardment (FAB), Tandem Mass Spectrometry (MS)), followed by chiral gas chromatographic procedures for absolute configuration assignments (all l-amino acid units). Experimental results inferred the heptapeptide skeleton and the high intensity of the molecular ion (base peak) plus the lack of terminal amino group protons in the ^1^H-NMR and IR spectra indicated a cyclic structure. The seven amino acids were readily identified by means of 2D-NMR techniques (Correlation Spectroscopy (COSY), Heteronuclear Multiple-Quantum Correlation (HMQC) spectroscopy, and Heteronuclear Multiple-Bond Correlation (HMBC) spectroscopy. Evidence for the linkage of the amino acid units was provided by HMBC correlations to suggest *cyclo*-[*trans*-Pro^1^-Ile^2^-*cis*-Pro^3^-Ile^4^-Phe^5^-*cis*-Pro^6^-Tyr^7^] as the structure of phakellistatin 1 [20]. In this review the numbering of amino acids in the peptide sequence do not follow that reported in the papers in order to be homogeneous in the current text. Moreover, although phakellistatins are very often shown as a mixture of conformers through the bonds Xaa-Pro, the conformer which showed prevalence is indicated in the text.

The structure (**1**, Figure 1a) was also confirmed by X-ray analysis (Figure 1b).

**Figure 2:**
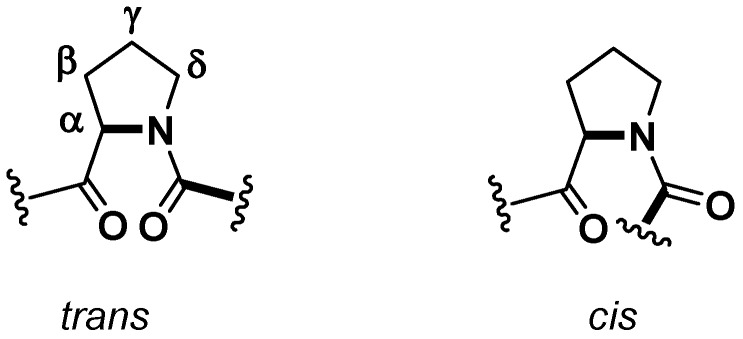
Possible conformers for the proline peptide bond.

A crucial aspect from a structural point of view, which could have some consequences on the biological activities [21], is the conformation of amide linkage. While, for most of the amino acids, the amide bond adopts a *trans* geometry, in the case of the Xaa-Pro bonds *cis* and *trans* isomers are energetically equivalent and, generally, in cyclic peptides the frequency of the *cis* geometry is higher (Figure 2). Cβ and Cγ ^13^C-NMR chemical shifts are indicative of the proline peptide bond conformations. A relatively large Δδ_cβ-cγ_ difference (approximately 8–10 ppm) suggests a *cis* isomer, while a smaller difference (approximately 3–4 ppm) points towards a *trans* one [19]. In this case both ^13^C-NMR and X-ray determination confirmed a *trans*, *cis*, cis geometry of the peptidyl-prolyl bonds for the proline named Pro^1^, Pro^3^, and Pro^6^, respectively.

The strong cell growth inhibitory activity (P388 murine leukemia, ED_50_ 7.5 μg/mL) of phakellistatin **1**, prompted a meticulous chemical investigation on different species of the *Phakellia* genus and, in twenty years, 18 prolinated homodetic cyclopeptide analogues of phakellistatin **1** (**1**) (commonly referred as phakellistatines) have been isolated and tested.

**Figure 3:**
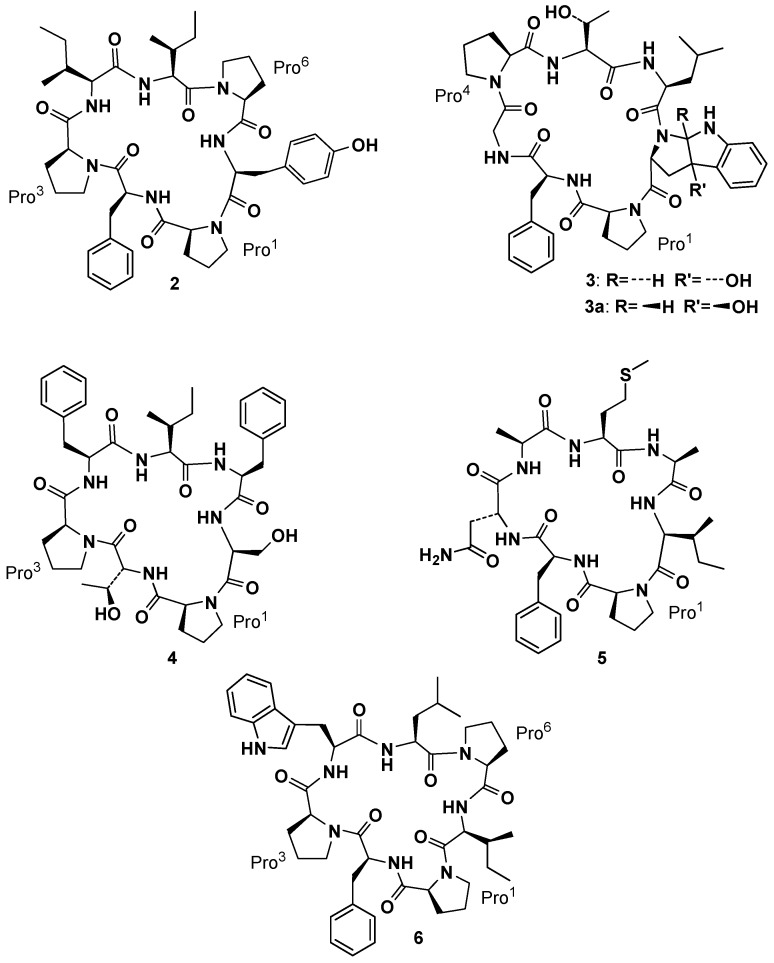
Phakellistatins **2**–**6**.

After discovering phakellistatin **1**, Pettit and co-workers isolated phakellistatin **2** (**2**, Figure 3) from *Phakellia carteri* (class *Demospongiae*, order *Axinellida*) [22]. Its structural elucidation through high-field 2D-NMR was initially complicated by the presence of two conformers in CDCl_3_ and CD_2_Cl_2_, but analysis in CD_3_OD, where only one conformer appeared, allowed, along with HR-FABMS techniques, to assign the structure as *cyclo*-[*cis-*Pro^1^-Phe^2^-*cis-*Pro^3^-Ile^4^-Ile^5^-*cis-*Pro^6^-Tyr^7^]. This cyclic heptapeptide exhibited significant cell growth inhibitory properties against murine P388 lymphocytic leukemia (ED_50_ 0.34 µg/mL) and a panel of human cancer cell lines. Soon after *Phakellia carteri* offered to Pettit’s group two new cycloheptapeptide, phakellistatin **3**, [*cis-*Pro^1^-Phe^2^-Gly^3^-*trans-*Pro^4^-Thr^5^-Ile^6^-*trans*-photo-Trp^7^] and its isomer isophakellistatin **3** (**3** and **3a**, Figure 3), containing unusual amino acid units, identified as *trans* and *cis*-3a-hydroxyl-1,2,3,3a,8,8a-hexahydropyrrolo[2,3-*b*]indole 2-carboxylic acid, respectively, and named by the authors, *trans*- and *cis*-photo Trp. Those residues, apparently derived from photoxidation of tryptophan, were never observed before. Interestingly, only phakellistatin **3** (and not its isomer **3a**) showed a significant P388 (ED_50_ 0.33 µg/mL) inhibitory activity. Isophakellistatin **3** (**3a**) X-ray crystal structure determination confirmed the assignment [23]. Bioassay guided (murine P388 lymphocytic leukemia cell lines) separation of the Chuuk Archipelago marine sponge *Phakellia costata* afforded phakellistatins **4**–**6** (**4**–**6**, Figure 3) [24,25,26]. Structural elucidation by high-field 2D-NMR and HR-FABMS techniques led to *cyclo*-[*cis-*Pro^1^-d-Thr^2^-*cis-*Pro^3^-Phe^4^-Ile^5^-Phe^6^-Ser^7^] as the assignment for **4**, *cyclo*-[*cis-*Pro^1^-Phe^2^-d-Asn^3^-Ala^4^-Met^5^-Ala^6^-Ile^7^] as the assignment for the principal solution conformation in CD_3_CN–CD_3_OD (3:1) for **5** and *cyclo*-[Pro^1^-Phe^2^-Pro^3^-Trp^4^-Leu^5^-Pro^6^-Ile^7^] as the assignment for **6**. At this stage the systematic occurrence of Pro residues in such compounds suggested the crucial role of peptidyl-prolyl bond geometry in the three-dimensional structure and, as a consequence, in their biological activities [26].

**Figure 4:**
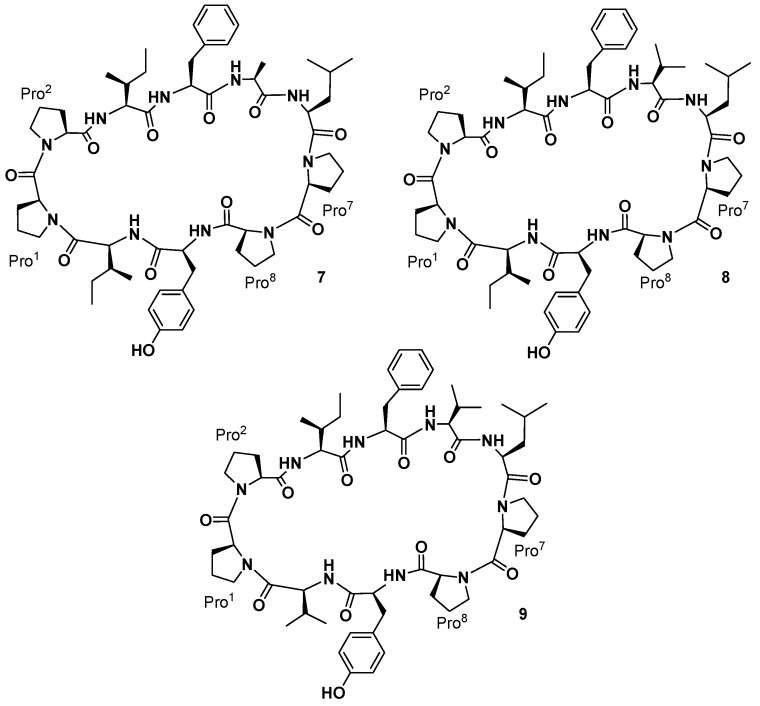
Phakellistatins **7**–**9**.

**Figure 5:**
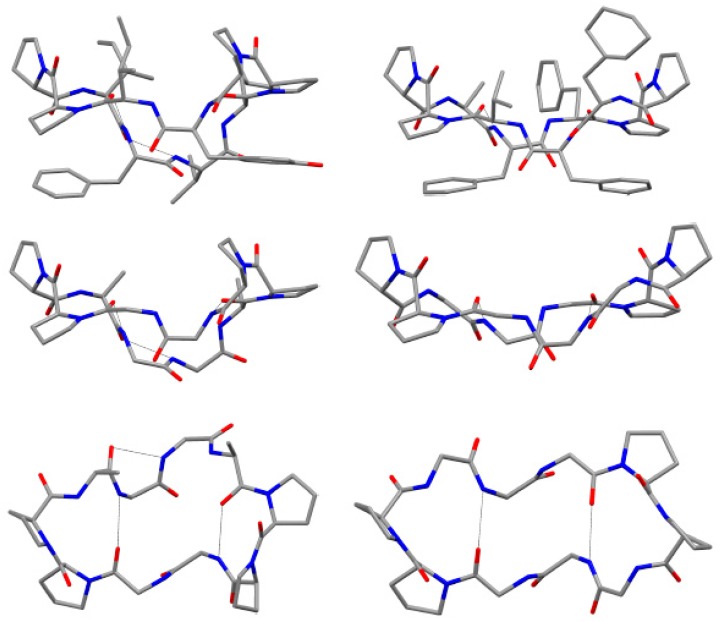
X-ray crystal structures of the cyclic peptide backbones (side view and top view) for phakellistatin **8** (left side) and antamanide (right side), showing intramolecular hydrogen bonds.

Pettit and co-workers also reported the isolation of three cyclic decapeptides, phakellistatins **7**–**9** (**7**–**9**, Figure 4), from the marine sponge *Phakellia costata* [27]. Phakellistatins **7**–**9** (**7**–**9**) are the first cyclic decapeptides isolated from a marine sponge with a cancer cell growth inhibitory activity (P388 ED_50_ 3.0, 2.9, and 4.1 μg/mL, respectively). The structure of phakellistatin **7**–**9** (**7**–**9**) was reported as *cyclo*-[Pro^1^-*cis*-Pro^2^-Ile^3^-Phe^4^-Ala^5^-Leu^6^-Pro^7^-*cis-*Pro^8^-Tyr^9^-Ile^10^], *cyclo*-[Pro^1^-*cis*-Pro^2^-Ile^3^-Phe^4^-Val^5^-Leu^6^-Pro^7^-*cis-*Pro^8^-Tyr^9^-Ile^10^], and *cyclo*-[Pro^1^-*cis*-Pro^2^-Ile^3^-Phe^4^-Val^5^-Leu^6^-Pro^7^-*cis-*Pro^8^-Tyr^9^-Val^10^], respectively. 2D-NMR spectra were recorded in CD_3_OD for phakellistatin **8** (**8**) and **9** (**9**), where essentially one conformational form was present (different conformational forms appeared in CDCl_3_ and CD_3_CN solvents). For phakellistatin **7** (**7**) the spectral analysis was conducted in CDCl_3_, where only one conformer appeared. Phakellistatin **7** (**7**) differs from the remaining two because in its sequence, an alanine is present (Ala-5). This is replaced by a Val residue in phakellistatin **8** (**8**) and phakellistatin **9** (**9**). Those last two secondary metabolites differ for an Ile-10/Val-10 substitution. *Cis-*amide bonds at CO(Pro^1^)/N(Pro^2^) and CO(Pro^7^)/N(Pro^8^) were confirmed by cross-peaks for H_α_(Pro^l^)/H_α_(Pro^2^) and H_α_(Pro^7^)/H_α_(Pro^8^) in all spectra, using Rotating frame nuclear Overhauser Effect Spectroscopy (ROESY). Further evidence for the *cis* geometries were obtained by the ^13^C chemical shift differences (Δδ_Cβ-Cγ_ 8.19–9.28 ppm) of the β and γ carbons of Pro^2^ and Pro^8^ residue. The l-configuration of the amino acid residues was determined by GC analysis of *N*-pentafluoropropyl isopropyl ester derivatives of the respective propionic acid-hydrochloric acid hydrolysates. Interestingly, although there are structural similarities and comparable activities against P388 cell in vitro, phakellistatin **7**–**9** showed significant differences against sixty human cancer cell lines from the U.S. National Cancer Institute panel [28]. Two years after the isolation, the X-ray crystal structure elucidation of phakellistatin **8** was reported. The solid-state structure of phakellistatin **8** was compared with that one of the well-known decapeptide antamanide, isolated from the extracts of the poisonous mushroom *Amanita phalloides* and showing a prophylactic protection from the lethal effects of the phallotoxins present in the same mushroom and an immunosuppresive activity (Figure 5) [28]. These two cyclopeptides, apparently phylogenetically very distant, present some structural similarities (e.g., the two *cis* Pro-Pro segments and the hydrogen bonds network). The chemical structure proposed by NMR was confirmed by diffractometric studies. All peptide bonds proved to be *trans*, with the exception of the *cis* at Pro^1^-Pro^2^ and Pro^7^*-*Pro^8^ linkages, as observed in solution. These residues are involved in two major turns inducing an elongated, saddle-like conformation, comparable to that assumed by the uncomplexed antamamide, which contains four proline units in the opposite sides of the macrocycle. This conformation in antamanide seems prodromal to cation complexation. The authors suggested a possible similar complexation ability for phakhellistatin **8**. This aspect could be a crucial point to understand the biological activities, considering the well-known correlation between complexation abilities and the bioactivities of such cyclic peptides [29]. Both compounds share further similarities: proline rings not only induce turns, but seem to determine the orientation of some side chains, the aromatic residues and the pyrrolidine ring are spatially close, and, finally, in the solid-state, a channel, occupied by the polar crystallization solvents (water and methanol), is present in phakellistatin **8**. Computational studies on the possible conformation of phakellistatin **8** in water solution, also suggested the possibility of an extended form (respect to that observed in the crystalline state), due to a transition of Tyr^9^ main-chain dihedral angles and the loss of the Pro^7^-Tyr^9^ staking [30].

**Figure 6:**
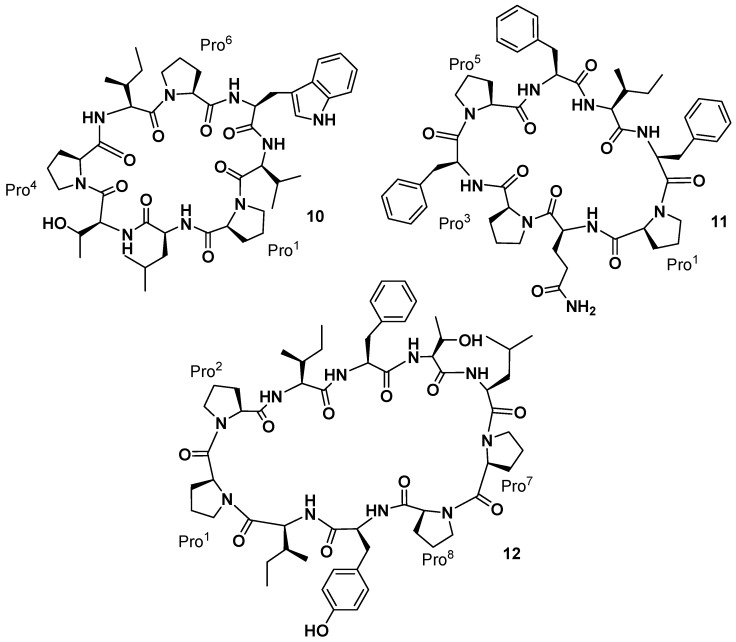
Phakellistatins **10**, **11**, and **12**.

The yellow-orange sponge *Phakellia* sp. (class *Demospongiae*, order *Axinellida*), collected in 1986–1987, in the Federated States of Micronesia (Chuuk), also afforded two new cyclic octapeptides that significantly inhibited the growth of the murine P-388 lymphocytic leukemia (ED_50_ values of 2.1 and 0.20 µg/mL, respectively) and human cancer cell lines [31]. The structure of these new marine sponge constituents, named phakellistatin **10** and **11** (**10** and **11**, Figure 6), were established using extensive tandem MS/MS and high-field (500 MHz) 2D, ^1^H-, and ^13^C-NMR analyses. The amino acid sequence of phakellistatin **10** (**10**) was determined to be *cyclo*-(*trans-*Pro^1^-Leu^2^-Thr^3^-*trans-*Pro^4^-Ile^5^-*trans-*Pro^6^-Trp^7^-Val^8^), and that of phakellistatin **11** (**11**) to be *cyclo*-(*cis-*Pro^1^-Gln^2^-*trans-*Pro^3^-Phe^4^-*cis-*Pro^5^-Phe^26^-Ile^7^-Phe^8^). The chemical shift difference (Δδ 4.11 ppm) between the β and γ carbons in Pro^3^ of phakellistatin **11** indicated a *trans* Gln^2^-Pro^3^ amide bond. All of the amino acid units (except Trp, which was not determined) were found to correspond to the l-configuration. In 1998 the two new compounds were patented for the treatment of one or more neoplastic diseases [32]. Indeed, phakellistatin **10** showed an LC_50_ of less than 10^−5^ M against the MDA-MB-435 breast cancer cell line and total growth inhibition for two breast cancers and two CNS cancer cell lines at a concentration of less than 10^−6^ M. Phakellistatin **11** presented a moderately lower level of in vitro activity as shown by an LC_50_ less than 10^−5^ M against the MDA-MB-435 breast cancer cell line and five other cell lines. It also achieved total growth inhibition for two breast cancers, one CNS cancer, one ovarian cancer, and one non-small cell lung cancer cell line at a concentration of less than 10^−7^ M. Interestingly, the structural relationship to hymenamides, proline-rich cyclopeptides [19], suggested a common microorganism genesis or the defensive role in closely-related marine sponges.

More recently, reinvestigation on trace fractions from the same sponge (collected in 1986–1987) led to the isolation by Pettit’s group of a new cancer cell growth inhibitory cyclodecapeptide (P388 ED_50_ 2.8 µg/mL) designated phakellistatin **12** (**12**, Figure 6), whose structure was assigned by NMR analysis as *cyclo*-[Pro^1^-*cis*-Pro^2^-Ile^3^-Phe^4^-Thr^5^-Leu^6^-Pro^7^-*cis-*Pro^8^-Tyr^9^-Ile^10^] [33]. Phakellistatin **12** has a peptide bonds arrangement similar to that present in the phakellistatin **8** (**8**). The only difference with **8** is the presence of a Thr^5^ instead of a Val^5^.

**Figure 7:**
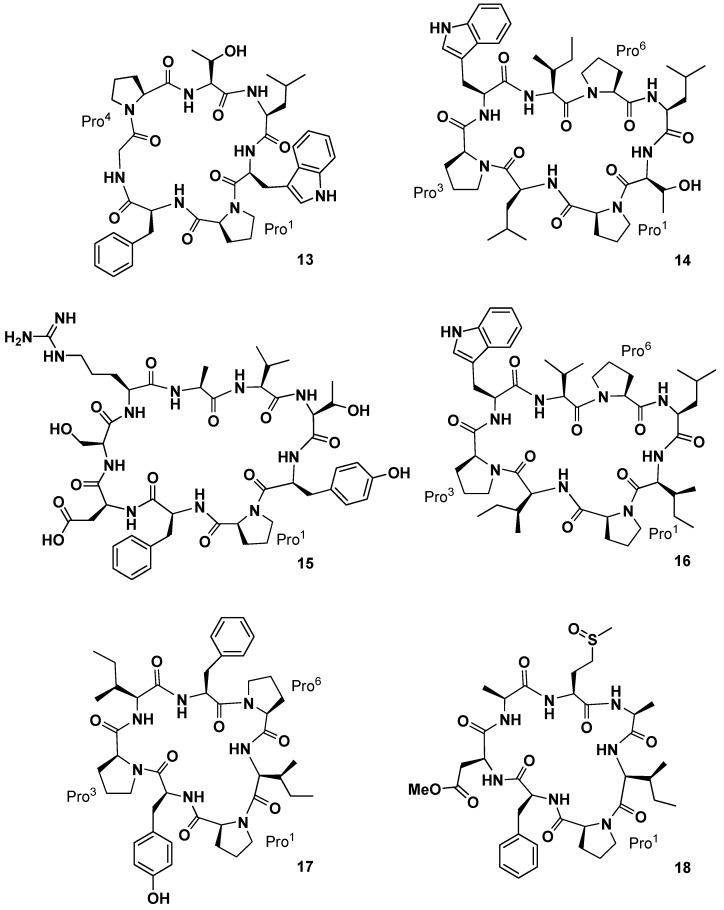
Phakellistatins **13**–**18**.

Yi and co-workers investigated the chemical constituents of the sponge *Phakellia fusca* Thiele, collected at Yongxing Island, in China [34]. A bioassay-directed separation of the crude EtOH extract of the marine organism yielded an active dichloromethane-soluble fraction containing the new cyclic heptapeptide phakellistatin **13** (**13**, Figure 7), cytotoxic against the human hepatoma BEL-7404 cell line with an ED_50_ < 10^−2^ µg/mL. The structure was assigned as *cyclo*-(Pro^1^-Phe-Gly-Pro^4^-Thr-Leu-Trp) on the basis of MS, UV, IR, and high-field NMR (600 MHz) analysis. A pharmacokinetic study on the determination of phakellistatin **13** in rat plasma, using liquid chromatography/tandem mass spectrometry, has been also reported [35]. From the same sponge, the Chinese group subsequently isolated four new cyclopeptides of different size, named phakellistatin **15**–**18** (**14**–**17**, Figure 7) [36]. Structural elucidation studies by High-Resolution Electrospray Ionization Mass Spectrometry (HR-ESIMS), NMR, and Matrix-Assisted Laser Desorption/Ionization (MALDI)-TOF/TOF sequence analysis showed for phakellistatins **15** and **17** (**14** and **16**) a structural relationship to hymenamide H (Pro-Trp-Val/Ile-Leu-Thr/Ile-Pro-Leu/Ile, analogous sequences) and for phakellistatin **18** (**17**) the same residues as those of phakellistatins **1** and **2**, but a different sequence. Phakellistatin **16** (**15**) is the first example of *n*BuOH-soluble natural phakellistatin extract of the genus *Phakellia* due to the hydrophilic Arg, Asp, Ser, and Thr residues. All of the macrocycles appeared as multiple rotamers in the most common deuterated solvents with the exception of phakellistatin **15** (**14**). In particular phakellistatin 16 presented a major and a minor conformer, which differed in the geometry of the Tyr-Pro peptidyl bond. Interestingly, while phakellistatin **15** and **16** exhibited cytotoxicity against the P388 cancer cell line (IC_50_ 8.5 and 5.4 µM respectively), phakellistatins **17** (**16**) and **18** (**17**) showed no activity. Phakellistatin **14** (**18**, Figure 7) was the last cytotoxic cyclopeptide (P388 ED_50_ 5 µg/mL) of phakellistatin family isolated by the Petitt group from the *Phakellia* sp. sponge, collected from Chuuk, Federated States of Micronesia [37]. Structural elucidation afforded the sequence: *cyclo*-(Pro-Phe-βOMeAsp-Ala-Met(O)-Ala-Ile). βOMeAsp and Met(O) are unique residue for this class of compounds. It is interesting to note that this compound is a derivative of phakellistatin **5** (**5**) differing for the presence of Asn, instead of Asp-methyl ester, and the sulfoxymethionine residue.

## 3. Phakellistatins: Total Synthesis and Puzzling Evidence

Since the isolation of the first member of the phakellistatin family several research groups, intrigued by the potent cytotoxicity, decided to synthesize them in order to have sufficient amounts to pursue the investigation on the biological activities and confirm the structures. Beyond chemical and structural validation, there was another problem that deemed to be solved: the biological properties of synthetic compounds greatly differed from those isolated from the organisms.

Kessler and Mechnich reported the total synthesis of phakellistatins **2** (**2**) and **4** (**4**) [38]. The linear precursors, prepared by solid phase synthesis (through Fmoc strategy, *o*-chlorotrityl chloride as the solid support and *N*-[(dimethylamino)-1*H*-1,2,3-triazolo-[4,5-*b*]pyridin-1-ylmethylene]-*N*-methylmethanaminium tetrafluoroborate *N*-oxide (TBTU)/1-hydroxybenzotriazole (HOBt) as the coupling system), were cyclized between Ile^4^ and Ile^5^ for **2** and Pro^3^ and Phe^4^ for **4**. After removal of side chain protecting groups and purification, the synthetic cyclopeptides were tested on different tumor cell lines, resultingly inactive. Considering the different pattern observed by NMR in the same deuterated solvent they supposed the primary structure for **2** and stereochemical assignment for **4** to be incorrect. However, from the ^13^C-NMR chemical shift values reported for phakellistatin **2**, it seemed evident that the geometry of the peptide bonds Xaa-Pro^1,3,6^ were *cis*, *trans*, *trans* and, thus, different from the all-*cis* peptide bond linkages reported for the natural product. Later, Pettit and co-workers confirmed their structural assignment through a second synthesis [39]. This time the synthesis of the linear precursor was made in solution by means of (4 + 3) segment condensation followed by cyclization with TBTU as the coupling reagent. This alternative synthesis afforded the chemically-identical cyclic peptide, but not biologically comparable to the natural phakellistatin **2**. To be absolutely sure of the identity of the synthetic sample, the authors repeated the NMR analysis even using an equal mixture of synthetic and natural compounds. Considering the lower inhibition activity observed for the synthesized sample (ED_50_ 24 µg/mL versus 0.34 µg/mL), Pettit and coworkers raised the question of whether the natural specimen could be contaminated by an undetectable amount of an extremely potent cytotoxin.

Another hypothesis was the possible presence of undetected different *cis*/*trans* proline conformations that were able to induce high biologically activity. The fortuitous isolation of two distinct conformers of phakellistatin **2** from the Fijian marine sponge *Stylotella auratium* shed light on the complex conformational behavior of the cyclopeptide and, again, raised the question of the biological activities’ discrepancies between synthetic and natural products [40]. Jaspars and co-workers confirmed the structural assignment made by Petitt: the NMR data of the more polar conformer were identical with those reported in the original paper [22] and Pettit’s total synthesis [39]. The less-polar conformer showed the same sequence and absolute stereochemistry of the isolated phakillestatin **2** but NMR spectra were significantly different from those observed in CD_3_OD. Those marked differences were attributed to a dissimilar solution conformation. Accurate theoretical studies on the conformations of phakillestatin **2** in solution demonstrated the possibility for this cyclic peptide to assume two independent conformations differing with the presence of a hydrogen bond between Phe^2^-NH and Ile^4^-C=O. Cytotoxicity assays, performed on both conformers, were highly dependent on the used solvent and on the time the samples were left in solution before the tests. This evidence could explain the discrepancies observed for the biological activities of the synthetized products [40].

**Figure 8:**
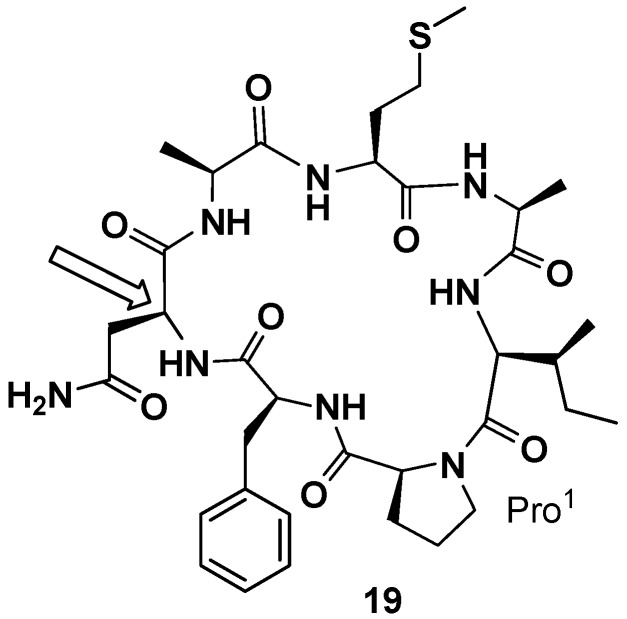
Revised structure of phakellistatin **5**.

In 2000 and 2001 Petitt and co-workers reported the total synthesis of phakellistatin **5** and **11**, respectively [41,42]. In both cases a peptide amide linker (PAL) resin was used and, proceeding from Fmoc-Asp-α-allyl ester and Fmoc-Glu-α-allyl ester, respectively, the linear precursors were synthetized. The allyl esters were removed under neutral conditions and cyclization, after deprotection of 9-fluorenylmethoxycarbonyl (Fmoc) group, was realized on the resin. The total synthesis of phakellistatin **5** allowed to correct the configuration assigned in the previous paper for Asn. The revised structure (**19**) is reported in Figure 8. Additionally, in those cases, the synthetic products were chemically, but not biologically, identical to the natural products and, resultantly, inactive. This time the hypothesis of the author to justify the lack of biological activity is the fortuitous coexistence, in the natural products, of a highly cytotoxic, spectroscopically undetectable, contaminant. The COMPARE analysis excluded the presence of metabolites already found in these sponges (halichondrin, halistatin, and spongistatin types) [41]. To support his claim, Pettit demonstrated the difficulty to evidence the presence, in an NMR sample, of very small quantities of a highly cytotoxic contaminant (100 µg) that can greatly influence the cell growth-inhibitory activity [42].

Gomez-Paloma and his research group found a synthetic route which yielded the cycloheptapetide phakellistatin **1** (**1**) and the cyclooctapeptide phakellistatin **10** (**10**) (together with yunnanis A and C) [43]. A Fmoc/*t-*Bu protection strategy was applied for the solid-phase synthesis of the linear precursors, using the 2-chlorotritylchloride resin as a solid support. Cyclization in solution using the efficient coupling reagent *N*-[(dimethylamino)-1*H*-1,2,3-triazolo-[4,5-*b*]pyridin-1-ylmethylene]-*N*-methylmethanaminium hexafluorophosphate *N*-oxide (HATU) afforded phakellistatin **1** as the predominant isomer characterized by *cis* geometries at the Tyr-Pro^1^, Ile-Pro^3^, and Phe-Pro^6^ peptide bonds (ROESY cross-peaks: Hα-Phe/Hα-Pro^6^, Hα-Tyr/Hα-Pro^1^, Hα-Ile/Hα-Pro^3^) and phakillestatin **10** as two products, the major one presenting an all-*trans* geometry at the Xaa-Pro bonds. Unfortunately, owing to the very low amount of the minor product, they were not able to identify the geometries at the Xaa-Pro bonds of the scarce geometrical isomer. While spectral data of the synthetic major conformer of phakellistatin **10** were superimposable with those reported for the natural product, contrary to what was stated by the authors, the synthetic phakillestatin **1** differed from the natural one (presenting a *trans* geometry at Tyr/Pro^1^ bond as was evident from Δδ_Cβ-Cγ_ (3.7 ppm) and X-ray crystal structure reported). Biological evaluation of the synthetic phakellistatin **1** and **10** against a minipanel of three cancer cell lines showed cell growth inhibitory activity with IC_50_ values always 100–1000-fold higher than their natural counterparts. Even if the synthetic phakellistatin **1** was different from the isolated one, the results observed for the synthetic phakellistatin **10**, albeit strange, was not unexpected, considering the results observed for the other synthetic cyclopeptides.

The group of the late Gomez-Paloma decided to further explore the structural and the biological aspects of these metabolites, facing the more complex synthesis of phakellistatin **7**, **8**, and **9** (**7**–**9**, Figure 4) with their unusual Pro-Pro sequences [44]. In the case of the chemical synthesis of phakellistatin **7**, only one conformational isomer was obtained and this was coincident with the natural one (*cis*-Pro^1^-Pro^2^, *cis*-Pro^7^-Pro^8^ linkages). The synthesis of phakellistatin **8** and **9** gave geometric isomers not chemically equivalent to the natural counterparts. The spectral data showed the presence of peptide geometries different from those of the isolated compounds. These results demonstrated the fact that, in cyclic compounds, prolines can deeply alter the geometric outcome of the synthetic product, stabilizing secondary structures different from those present in the extracts from the marine organisms.

Not surprisingly, the biological test on a mini-panel of three cancer cell lines showed, for the four synthetic geometrical isomers of phakillestatin **8** and **9**, lower cell growth inhibitory activity than the natural counterparts. Additionally, phakellistatin **7**, albeit chemical equivalent to the natural product, showed lower activity compared with the isolated congener. According to the authors the biological discrepancy could be ascribed to slight conformational differences between the natural and the synthetic products, difficult to detect by means of ROESY correlations. They suggested that the enzymatic machinery, occurring in the marine organism and responsible of the specific three-dimensional arrangement, could be hardly mimed by a synthetic cyclization step where more degrees of freedom are possible.

In the 2004 Van Vranken and co-workers, intrigued by the homology of phakellistatine **13** (**13**) peptide sequence with several known human proteins and by the potential biogenetic relationship with phakellistatin **3** (**3**), containing the 3a-hydroxypyrrolidino [2,3-*b*]indoline (Hpi) moiety, reported the total synthesis of phakellistatine **13** and its oxidative cyclization to phakellistatine **3** and isophakellistatine **3** [45]. With their contribution they confirmed the absolute configuration of the stereogenic center of the Trp residue and proved that Trp-containing peptides can be oxidized to the corresponding Hpi-containing sequence. Macrocycle formation was performed on a linear peptide (obtained on solid-phase with the classical Fmoc-strategy) with the glycine at carboxy terminus using *N*-[(**1** *H*-benzotriazol-1-yl) (dimethylamino)-methylene]-*N*-methylmethanaminium hexafluorophosphate *N*-oxide (HBTU)/HOBt. With phakellistatin **13** in hand, the oxidation was investigated and a 1:1 mixture of phakellistatin **3** and isophakellistatin **3** was obtained in a combined 20% yield by O_2_ photoxidation in the presence of a sensitizer, followed by reduction of the hydroperoxide with Me_2_S. A synthesis of phakellistatin **13**, totally performed in solution by combination of two peptide segments was reported by Zhou and coworkers later [46].

No biological test was performed on the synthetic cyclodecapeptide phakellistatin **12** (**12**), whose preparation was reported by the Shadeen’s group [47]. The total synthesis, achieved after cyclization of a linear precursor, prepared on solid-phase with an Fmoc-protocol and using the Kenner’s *N*-acyl “safety-catch” linker, afforded a unique isomer and confirmed the correct structure assignment of the natural product. More recently the same group reported the solid-phase synthesis of phakellistatin **15** (**14**), using an analogous synthetic strategy [48]. In this case the synthetic product, spectrally identical to the reported natural compound, proved to be inactive on human glioblastoma (U-87), pancreatic cancer (PSN-1), and human non-small cell lung cancer (NCI-H460) cells.

**Figure 9:**
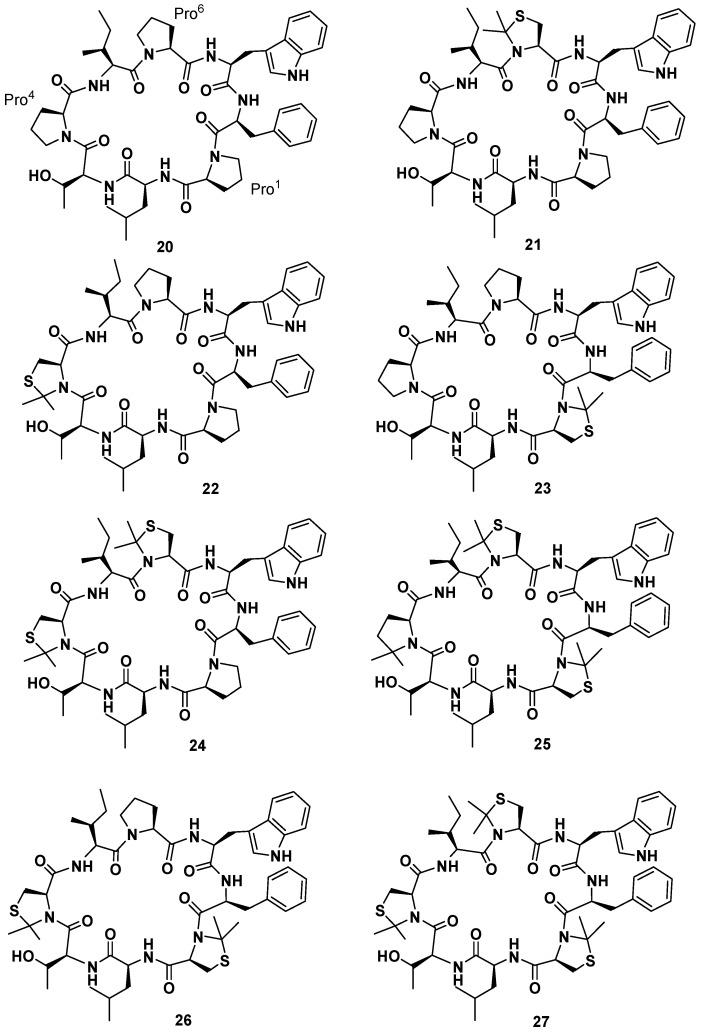
Phakellistatin **19** (**20**) and its analogues.

The latest isolated member of the class of phakellistatins is the phakellistatin **19** (**20**, Figure 9), first reported by Albericio et al. Phakellistatin **19** is a cyclooctapeptide reminiscent of phakellistatin **10** (**10**) for its amino acid sequence and structure, containing a high number of non-polar amino acids, and presenting a residue of Phe instead of the amino acid Val [49]. Synthetic phakellistatin **19** was found to be chemically, but not biologically, identical to the natural product, like the other members of its family. HPLC-PDA analyses of the synthetic, natural, and mixture samples showed that each specimen had the same retention time. In addition, ^1^H-NMR studies performed in deuterated methanol gave two patterns of signals perfectly superimposable for the synthetic and natural compounds, and the one performed in DMSO-*d*_6_ on synthetic phakellistatin **19** gave well resolved signals corresponding to just one conformer or a mixture of conformers in fast equilibrium on the NMR time scale. The ROESY spectrum cross-peaks H_α_-Phe^7^/H_δ_-Pro^1^, H_α_-Thr^3^/H_δ_-Pro^4^ H_α_-Ile^5^/H_δ_-Pro^6^, provided evidence of the *trans* geometry of all of the Xaa-Pro amide bonds. This was also supported by the small difference between β and γ ^13^C NMR chemical shifts of prolines (Pro^1^ Δδ_Cβ-Cγ_ = 5.01 ppm; Pro^4^ Δδ_Cβ-Cγ_ = 4.38 ppm; Pro^6^ Δδ_Cβ-Cγ_ = 3.78 ppm). Despite the chemical equivalence, biological evaluation of the synthetic phakellistatin **19** against a mini-panel of three cancer cell lines (human breast adenocarcinoma, human lung adenocarcinoma, and human colon adenocarcinoma) did not show any cell growth inhibitory activity, whereas its natural counterpart resulted to inhibit cellular growing with GI_50_ values comprised between 4.41 × 10^−7^ and 5.15 × 10^−7^ M. pH dependent conformational studies were realized for an H_2_O soluble analogue of **20**, containing Orn at Leu position. At pH 5.95 and 8.12 the NMR studies proved that no conformational variation of the *trans* geometries at the prolyl bonds of Xaa-Pro occurred. Moreover, in order to investigate the correlation between the biological activities and the *cis*/*trans* geometry at Xaa-Pro linkages, considering that a conformational change could play an important role in cytotoxicity [19], the synthesis of seven phakellistatin **19** constrained analogues was realized (**21**–**27**, Figure 9) [49]. An efficient tool to constrain the prolyl peptide bond into a *cis* conformation is represented by its substitution with a 2,2-dimethylated residue: Cys(Ψ^Me,Me^pro) [50]. Recently, the incorporation of the mimic 2,2-dimethyl-1,3-thiazolidine-4-carboxylic acid at position 7 of the hormone oxytocin bonded to Cys^6^ through a peptide linkage (exclusively existing in the *trans* conformation) yielded an analogue that showed a 92%–95% *cis* conformation and no antagonistic activity [51]. As a consequence a small library of seven cyclooctapeptides replacing Pro residues with Cys(Ψ^Me,Me^pro) was designed, prepared, and tested. The synthetic strategy had to be different from that applied for the natural product, in fact the steric hindrance of the Cys(Ψ^Me,Me^pro) needed the solution synthesis of the Cys(Ψ^Me,Me^pro) containing dipeptides. Biological data evidenced a correlation between the number of Cys(Ψ^Me,Me^pro) units and the cytotoxic activity. Moreover, they showed that **21**, in which Cys(Ψ^Me,Me^pro) replaces Pro^6^, was the most active monosubstituted analogue. Furthermore, an increasing bioactivity of the compounds was observed with increasing numbers of Cys(Ψ^Me,Me^pro) residues in phakellistatin 19, reaching the highest value for the analogue **27**. NMR analysis of the library revealed that the incorporation of more Cys(Ψ^Me,Me^pro) units also led to more conformationally-restricted peptides, inducing the *cis* geometry at the Xaa^i+1^-Cys(Ψ^Me,Me^pro)^i^ linkages. Indeed, while **21** showed a complex mixture of conformers in slow equilibrium on the NMR time scale, due to the *cis*-*trans* isomerization at the Pro bonds, **27** appeared as a major all-*cis* conformer. The above observations evidenced a correlation between the number of Cys(Ψ^Me,Me^pro) units and the amount of the *cis* conformation in the final peptides, which favors the cytotoxic activity. Ultimately, this result suggests a method to improve or modulate the biological activities of proline-rich cyclopeptides.

In conclusion the isolation of numerous compounds of phakillestatin family has led to interesting findings in the search for secondary metabolites of marine origin. The structural peculiarities, due to the presence of proline residues, the intriguing and steady biological activities observed, leave many questions still unresolved, both from a synthetic point of view, mainly related to the difficulty of obtaining the natural conformational isomers, and from a biological one, due to the difficulty to reproduce their cell growth inhibition activity of tumor cells. This interesting, and relatively small family of compounds leaves much room for further exploration and will lead to new insights in the realm of marine drugs.

## Figures and Tables

**Table 1:** Summary of reported data on phakellistatins.

Phakellistatin ^[a]^	Marine Sponge	Biological Activity ED_50_ (μg/mL)	Synthetic Studies (Reference Number)
1 (**1**)	*Phakellia costata* and *Stylotella aurantium*	7.50 ^[b]^	43
2 (**2**)	*Phakellia carteri*	0.34 ^[b]^	38, 39
3 (**3**)	*Phakellia carteri*	0.33 ^[b]^	45
iso 3 (**3a**)	*Phakellia carteri*	not active ^[b]^	45
4 (**4**)	*Phakellia costata*	0.32 ^[b]^	38
5 (**5**)	*Phakellia costata*	0.23 ^[b]^	41
6 (**6**)	*Phakellia costata*	0.18 ^[b]^	-
7 (**7**)	*Phakellia costata*	3.2 ^[b]^	44
8 (**8**)	*Phakellia costata*	2.9 ^[b]^	44
9 (**9**)	*Phakellia costata*	4.1 ^[b]^	44
10 (**10**)	*Phakellia* sp.	2.1 ^[b]^	43
11 (**11**)	*Phakellia* sp.	0.20 ^[b]^	42
12 (**12**)	*Phakellia* sp.	2.80 ^[b]^	47
13 (**13**)	*Phakellia fusca*	<10^−2 [c]^	45, 46
14 (**18**)	*Phakellia* sp.	5.0 ^[b]^	-
15 (**14**)	*Phakellia fusca*	7.8 ^[b,d]^	48
16 (**15**)	*Phakellia fusca*	5.6 ^[b,d]^; 14.8 ^[c,d]^	-
17 (**16**)	*Phakellia fusca*	not active ^[b,c]^	-
18 (**17**)	*Phakellia fusca*	not active ^[b,c]^	-
19 (**18**)	not reported	4.41 × 10^−7^; 4.62 × 10^−7^; 5.51 × 10^−7 [e]^	49

^[a]^ In parenthesis the adopted numbering of phakellistatins in the current review; ^[b]^ Cell growth inhibitory activity (P388 murine leukemia); ^[c]^ Cytotoxicity against human hepatoma BEL-7404 cell line; ^[d]^ Expressed as IC_50_ (μg/mL), compound concentration that produces 50% inhibition of biological activity; ^[e]^ Expressed as GI_50_, compound concentration that produces 50% of cell growth inhibition compared to control cultures of NSCLC (lung) A549, colon HT-29, and breast MDA-MB-231 cell lines, respectively.
